# Case Report: Acral vasculitis induced by Immune Checkpoint Inhibitors: a case series and literature review

**DOI:** 10.3389/fonc.2025.1537825

**Published:** 2025-05-09

**Authors:** Valérian Rivet, Benoit Guillon, Vincent Sibaud, Jérémie Dion, Andréa Pastissier, Karen Delavigne, Pierre Cougoul, Odile Rauzy, Thibault Comont

**Affiliations:** ^1^ Internal Medicine and Immunopathology Department, The Cancer University of Toulouse Oncopole, University Hospital Center of Toulouse, Toulouse, France; ^2^ Dermatology Department – Medical Oncology, The Cancer University of Toulouse Oncopole, University Hospital Center of Toulouse, Toulouse, France

**Keywords:** acral vasculitis, immune checkpoint inhibitors, immune-related adverse events, nivolumab, pembrolizumab, ipilimumab

## Abstract

**Introduction:**

Immune Checkpoint Inhibitors (ICIs) may cause various immune- related Adverse Events (irAEs). Of these events, vascular involvement is still considered an uncommon irAEs and generally concerns large or medium vessels. Acral small-vessel vasculitis can lead to severe digital necrosis.

**Case presentation:**

Herein, we present three cases after treatment with pembrolizumab, nivolumab, and combination nivolumab/ipilimumab for lung adenocarcinoma, renal cell carcinoma, and melanoma, respectively. Two patients had a Raynaud’s-like syndrome. All of them presented with digital ischemia of both hands and with severe acral necrosis in the first case. Management consisted in ICI discontinuation, high-dose steroids, and vasodilator agents with good evolution in the three cases. No rechallenge of ICI has been attempted.

**Discussion:**

We found 12 other cases in the literature review to build a cohort of 15 patients, mostly male with a median age of 60 years. Lung cancer and melanoma are the most common tumors. The most frequently used ICI was pembrolizumab. The median time to onset was 8 weeks. The main clinical presentation was a distal and painful necrosis mostly on thehands with bilateral involvement. Toes were affected in only two cases. All cases were severe features with grade ≥ 3. Eleven patients were treated with steroids and vasodilator agents. ICI was discontinued permanently in all patients.

**Conclusion:**

ICI-induced small-vessel vasculitis can lead to severe digital ischemia, often in males, and is preceded by a Raynaud’s-like syndrome with mostly bilateral and hand involvement. Data are still missing to optimize management of these kinds of patients.

## Introduction

Immune Checkpoint Inhibitors (ICIs), either alone or in combination, are now used in several advanced malignancies and may cause various immune-related Adverse Events (irAEs) that can affect virtually every organ system. Vascular involvement is still considered an uncommon immune side effect and was not described in early trials. In 2018, a systemic review evaluated 20 cases of vasculitis that occurred after ICIs (1). The main reported type was large-vessel vasculitis (Giant Cell Arteritis) and vasculitis of the central and peripheral nervous system. The predominant cancer was melanoma with a median time to onset of 3 months.

Most cases seemed to be resolved with high-dose systemic steroids and ICI discontinuation. For small vessel involvement, two cases of acral vasculitis are described: one after anti- programmed death-ligand 1 (anti-PD-L1) and one after anti-cytotoxic T-lymphocyte- associated protein 4 (CTLA-4) (1–3). Recently, more published studies have confirmed an association between ICIs and acral small-vessel vasculitis, which can lead to digital severe necrosis with poor response to glucosteroids and which requires surgical amputation (4, 5). The incidence of acral vasculitis seems very low, with only three cases found in 447 patients (0.007%) who developed connective tissue diseases after PD-1/PD-L1 treatment (4).

The pathophysiology is not fully understood, but increasing evidence suggests that the immune checkpoint plays a key role in immune and inflammatory homeostasis of the vasculature (1, 4, 5). ICIs enhance the immune system through blockage of costimulatory signal receptors, inducing hyperstimulation of the immune system against some antigens in healthy tissue and vessels. They can also increase levels of pre-existing autoantibodies (auto-ab) and of inflammatory cytokines, resulting in several irAEs, such as dysthyroidism, colitis, pneumonitis, and, rarely, myocarditis. Some authors have shown that the inhibition of the PD-1/PD-L1 axis induced T-cell hyperactivity in the vessel wall, as well as causing dendritic cell impairment and the production of cytokines and auto-ab that can promote vasculitis (1, 4).

Moreover, ICIs can cause endothelial insult, leading to both atherosclerosis lesions and a procoagulable state (4, 6). Finally, some authors have suggested a relationship to paraneoplastic acral vascular syndrome (PAVS), whose pathomecanism includes hyperviscosity, hypercoagulability, vasospasm, and spontaneous platelet aggregation (7, 8).

Nevertheless, ICI-induced-acral vasculitis (AV-ICI) is still poorly described in both diagnosis and treatment. We propose three more cases and a literature update.

## Cases presentation

### Case 1

**Figure 1:**
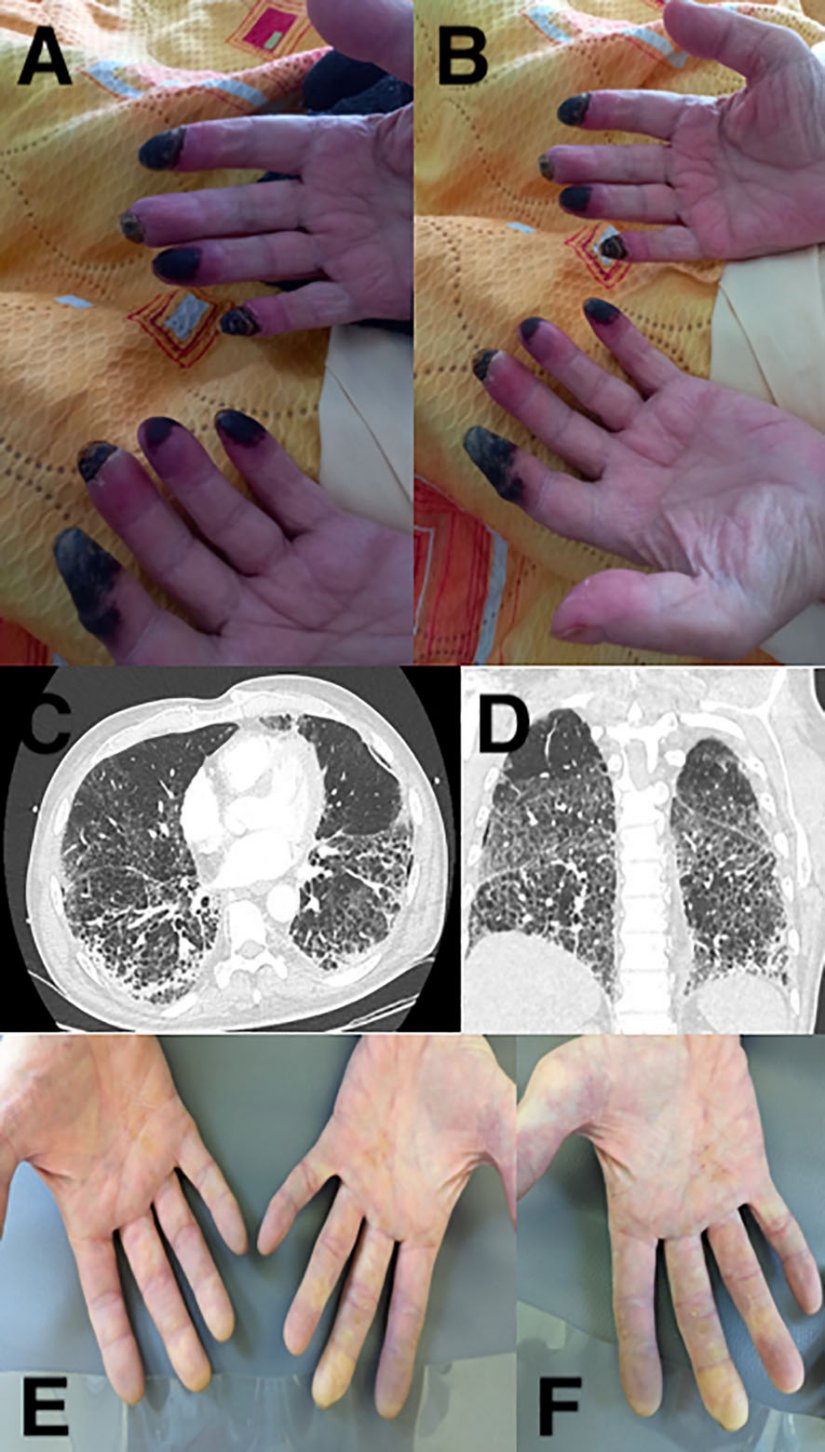
Main clinical and imaging features of the case series. Digital necrosis of case 1 **(A, B)**, diffuse interstitial pneumonia of case 1 **(C, D)**, and bilateral digital ischemia of both hands of case 2 **(E, F)**.

A patient in their 60s was initiated on combination chemoimmunotherapy with carboplatin, pemetrexed, and pembrolizumab for metastatic lung adenocarcinoma in June 2022. After four cycles, he was placed on maintenance therapy with pemetrexed and pembrolizumab. In November, after eight cycles of ICI, the patient suffered from a Raynaud’s-like syndrome with purple discoloration of all fingers of both hands, including the thumbs. He had no history of acrosyndrome or dysimmune disease but did have a history of smoking, which he had stopped in 2016. In December 2022, the ninth cycle was not completed because of the sudden appearance of painful digital necrosis on both hands (**Figures 1A, B**). Both chemo and immunotherapy were stopped and the patient was hospitalized. Physical examination showed distal gangrene (sparing the lower limbs) with perinecrotic erythema without any clinical signs of associated rheumatologic/vascular disease or other irAEs. Admission laboratory tests revealed a normal metabolic (including thyroid parameters) and coagulation profile. Immunological tests, including protein electrophoresis, cytoplasmic and perinuclear anti-neutrophil cytoplasmic antibody (ab), complete panel of scleroderma ab, rheumatoid factor, cryoglobulins, cryfibrinogens, anti-phospholipids, anti-extractable nuclear antigen, and anti-DNA ab, were all negative, except for antinuclear ab (ANAs), which were positive at 1:5120. Blood tests were also negative for hepatitis B/C and HIV and spot urine tests revealed no proteinuria. In addition, arterial Doppler of the upper limbs and an echocardiography did not reveal any significant abnormality. Nail fold capillaroscopy showed peri-capillary edema without any associated changes (including a lack of identified megacapillaries). Skin punch biopsy was not performed because of the very high risk of worsening the lesions. A diagnosis of AV-ICI was nonetheless retained considering the clinico-biological features and the absence of a differential diagnosis in our exhaustive comprehensive work-up. ICI perfusions were discontinued.

Prednisone was initiated (1 mg per kg daily for four days), which unfortunately caused worsening of both pain and cutaneous involvement. So, the patient started a three-day course of intravenous (IV) methylprednisone (500 mg per day), calcium blockers, and iloprost to stabilize the extension of the digital necrosis and improve pain. Corticosteroids (CS) were tapered to 1 mg/kg/d. A few days later, he suddenly suffered from respiratory failure that required mechanical ventilation. A CT-scan revealed both A right proximal pulmonary embolism and diffuse interstitial pneumonia (DIP) (**Figures 1C, D**). Steroid treatment was increased to 2 mg/kg/d without improvement on respiratory parameters. New blood tests did not find any abnormalities once again. Finally, a rescue treatment of tocilizumab infusion (8 mg per kg, IV) was attempted. Progressive recovery led to extubating, weaning of oxygen therapy, and complete stabilization of skin lesions as drying necrosis. A TEP-scan found neoplastic progression of both the lung and metastatic target. After more than two months, the patient was discharged. Contraindication was retained for ICI treatments. Secondarily, he benefited from surgical amputation. Six months later, there has been no sign of respiratory or cutaneous worsening despite cortisone withdrawal.

### Case 2

A man, about 65 years old, with metastatic renal cell carcinoma was treated with nivolumab combined with cabozantinib (an inhibitor of both met receptor tyrosine kinase and the VEGF receptor 2). He had no history of acrosyndrome, dysimmune disease, or smoking. Between September 2022 and February 2024, he received 24 cycles (nivolumab 240 mg every 2 weeks with cabozantinib 40 mg per day). In November 2023, a CT-scan showed a partial response both in primitive lesion and metastatic lymph nodes. After 15 cycles, a systemic blood test revealed grade 3 cytolytic hepatitis and peripheral hypothyroidism, resolved after ICI discontinuation and six weeks of steroids and hormone supplementation. In March 2024, he was hospitalized for painful discoloration of all fingers on both hands. On examination, we found purple discoloration of the bilateral fingers (including the thumbs but sparing the others extremities) without ulceration or necrosis (**Figures 1E, F**).

Routine blood chemistries, coagulation profiles, and immunological tests were normal. Upper extremity arterial Doppler ultrasound showed only partial and chronic thrombosis of the right ulnar arterial. Nail fold capillaroscopy and skin biopsy were not performed in this patient because of the quick improvement of clinical features after the start of treatment. Both nivolumab and cabozantinib were stopped. The patient started prednisone (1 mg/kg/d). After two days without improvement, iloprost was suggested. After two more days, both acral ischemia and pain clearly improved with complete response and the patient was discharged after eight days. Unfortunately, a new CT-scan revealed worsening of adenopathy and pulmonary metastatic involvement. Because of this oncologic progression and the immune-related vascular toxicity, it was decided to stop ICI perfusions and switch to single-agent lenvatinib (a multi-tyrosine receptor inhibitor). After 3 months, a new CT-scan showed stable oncologic disease in both primitive and metastatic targets, without any recurrence of digital ischemia or vascular involvement.

### Case 3

A 60-year-old man, without any smoking history, dysimmune disease, or acrosyndrome, was treated for advanced melanoma with neoadjuvant combination by nivolumab and ipilimumab (respectively 1 mg/kg and 3 mg/kg every 3 weeks) in July 2021. Five days after the second perfusion, he developed a sudden severe Raynaud’s-like syndrome associated with paresthesia and cyanosis mostly on both second fingers and the fourth and fifth fingers on the left hand. He did not have myalgia or motor deficiency. In the vascular department, his routine blood chemistries, viral serologies, and coagulation profile were normal.

Immunological tests found only specific anti-Jo1-ab with negative screening of ANA. Bilateral upper extremity arterial Doppler ultrasound was normal and nail fold capillaroscopy showed only non-specific abnormalities. A thoracic CT-scan did not show any vascular abnormalities or interstitial pneumonia. Both elevation on troponin I (194 ng/ml, N < 14) and CPK levels (2156 UI/l, N < 190) were highlighted. Cardiac IRM was normal but an endomyocardic biopsy showed diffuse lymphocytic infiltration and confirmed the immune-related myocarditis. The association of the severe Raynaud’s phenomenon, elevated CPK, myocarditis, and positive anti-Jo1 ab suggested an anti-synthetase syndrome triggered by ICIs. An immune-related acral vasculitis was confirmed without histological features in the absence of a differential diagnosis. ICIs were stopped. The patient received three methylprednisone IV perfusions (1g/d) and then prednisone (2 mg/kg) before progressively tapering off, as well as iloprost for 28 days. The patient had complete recovery of both cardiac and vascular involvement. Oncologic follow-up found a complete response of the melanoma. After 6 months, a new tumor assessment revealed a persistent complete metabolic response and a rechallenge of ICI has not yet been carried out. The patient had no recurrence of acral vascular manifestations despite progressive cortisone withdrawal.

## Discussion

Vasculitis has not been documented as an irAE after anti-PD-1/L-1 and/or anti-CTLA4; it is seen in <1% of cases, with large-vessel involvement in most of them (1, 5). Acral vasculitis seems to be an extremely rare manifestation of ICI therapy, with often severe clinical features and still very little data about their management. We described three new cases of small-vessel involvement after ICI. We also found 12 other cases in an update of the literature review to form a cohort of 15 different patients (**Table 1**).

**Table 1:** Previously published and current case of Immune Checkpoint Inhibitor-related (ICI) acral vasculitis.

Author ^Ref^ (year)	Age/ Gender	Cancer	ICI (ICI-associated therapy)	Onset* (number of perfusion)	Main skin lesions	Systemic symptoms/other irAEs	Immunological findings	Grade**	Treatment/ Outcome of the irAEs/ Rechallenge
Rivet V, et al. (2025)	60/ Male	Lung ADK	Pembrolizumab (carboplatin/ pemetrexed)	24 (8)	Raynaud-like syndrome and then bilateral severe digital necrosis of both hands	Severe DIP	ANAs (titer 5120, speckled pattern)	4	ICI discontinuation; prednisone; methylpredinisolone; calcium blockers; iloprost; tocilizumab; late surgical amputation PR; no rechallenge
	65/ Male	RCC	Nivolumab (cabozantinib)	68 (24)	Painful bilateral ischemia of all fingers without necrosis	Cytolytic hepatitis; peripheral hypothyrodism	None	3	ICI discontinuation; prednisone; iloprost; CR; no rechallenge
	60/ Male	Melanoma	Nivolumab + Ipilimumab	4 (2)	Bilateral Raynaud-like syndrome with severe cyanosis of fingers of both hands	Myocarditis	Anti-Jo1 (negative ANAs)	3	ICI discontinuation; methylpredinisolone; iloprost; CR; no rechallenge
Yohannan B, et al. (5) (2023)	60/ Male	Lung ADK	Pembrolizumab (carboplatin/ pemetrexed)	8 (4)	Bilateral Raynaud-like syndrome, acral necrosis of fingertips	Esophagitis; peripheral neuropathy	ANAs (titer 640, speckled pattern)	4	ICI discontinuation; methylpredinisolone; aspirin, sildenafil, nitropaste, prostacycline; worsening of irAE; no rechallenge
	72/ Male	ORL SCC	Pembrolizumab (carboplatin, paclitaxel)	24 (8)	Acral necrosis of left arm, right foot, ear and nose	None	None	4	ICI discontinuation; supportive care; no rechallenge
O’Connor P, et al. (9) (2020)	45/ Male	TNBC	Pembrolizumab (carboplatin, docetaxel)	18 (6)	Erythema, edema and tender right 3nd digit and onycholysis, Raynaud-like syndrome	None	ANAs (titer 160, speckled pattern), anti-RNA pol-III	3	ICI discontinuation; anticoagulation; calcium blockers, prednisone; sildenafil; PR; no rechallenge
Franco, F et al. (10) (2019)	46/ Male	RCC	Nivolumab	8 (4)	Pain and ischemia of the all fingers of both hands	None	None	3	ICI discontinuation; methylpredinisolone; anticoagulation; aspirin; MMF, iloprost; CR; no rechallenge
Khaddour, K, et al. (4) (2019)	68/Female	Lung ADK	Pembrolizumab	25	Bilateral Raynaud-like syndrome, acral necrosis of all fingers	None	ANAs (titer 80, speckled pattern)	4	ICI discontinuation (late); calcium blockers; prednisone; sympathectomy; worsening of irAE requiring surgical amputation; no rechallenge
Comont T, et al. (7) (2018)	66/ Male	Urothelial bladder cancer	Tremelimumab + Durvalumab	8	Periungual skin necrosis of several digits of both hands	None	ANAs (titer 5200, speckled pattern)	3	ICI discontinuation; prednisone; CR; no rechallenge
Padda A et al. (2) (2018)	52/ Male	Melanoma	Ipilimumab	3 (2)	Raynaud-like syndrome, subungual necrosis on several upper and lower limb digits, rash	Myalgia, athralgia, vision changes, jaw pain, DIP	None	3	ICI discontinuation; methylpredinisolone, prednisone, calcium blockers, nitropaste, prostacycline, botulism toxin, sildenafil; rituximab; worsening of irAE requiring surgical amputation; no rechallenge
Narvaez J, et al. (11) (2018)	49/ Male	Lung ADK	Nivolumab	2 (1)	Ulceronecrotic lesions of several fingers of both hands	None	None	3	ICI discontinuation; aspirin; prostacyclin; calcium blockers and bosentan; CR; no rechallenge
Leburrel S, et al. (3) (2018)	60/ Male	Melanoma	Anti-PDL1 (anti-MEK/anti-BRAF)	8	Cyanosis and necrosis of 3 fingers and the heels	Arthralgia, xerostomia, paresthesia of the feet and interstitial pneumonia	ANAs (titer 160, speckled pattern), anti-SSa; cryoglubulinemia (type III)	3	ICI discontinuation; prednisone; calcium blockers; iloprost and aspirin; PR; no rechallenge
Gambicher, T, et al. (8) (2017)	60/ Male	Melanoma	Nivolumab + Ipilimumab	3	Subungual necrosis on the fingertips of both hands, severe gangrene	None	None	4	ICI discontinuation; prednisolone; prostacycline; methylprednisone; nitroglycerin; iloprost; calcium blockers; worsening of irAE requiring surgical amputation; no rechallenge
Thoreau B, et al. (12) (2016)	73/ Male	Melanoma	Pembrolizumab	26	Acute ischemia of the left toes	None	None	4	ICI discontinuation; anticoagulation; iloprost; aspirin; worsening of irAE requiring surgical amputation; no rechallenge
Takada K, et al. (13) (2021)	60/ Male	Lung carcinoma	Pembrolizumab (carboplatin, docetaxel)	2 (1)	Raynaud’like syndrome, bilateral acral necrosis of fingers and toes	Acute renal failure	None	4	ICI discontinuation; prednisone; vasodilatator agents CR; no rechallenge

*Weeks between initiation of immunotherapy and the diagnosis of acral vasculitis.

** Grade of the vascular irAEs according to the CTCAE version 5.0.

ICI, Immune Checkpoint Inhibitor; irAEs, immune-related Adverse Events; ADK, Adenocarcinoma; DIP, diffuse interstitial pneumonia; PR, partial response; ANAs, antinuclear antibodies; RCC, renal cell carcinoma; SCC, squamous cell carcinoma; TNBC, triple-negative breast carcinoma; MMF, mycophenolate mofetil; CR, complete response; CTCAE, Common Terminology Criteria for Adverse Events.

The median age of this cohort is 60 (min-max 45-73; SD 8,95), with a clear male predominance (only one case involved a woman) (6,7%) (**Table 1**) (2–5, 7–13). Lung cancer is the most common tumor (five cases, 33%), especially lung adenocarcinoma with melanoma (five cases, 33%). The most frequently used ICI is pembrolizumab (seven; 47%), often associated with chemotherapy. A combination of ICIs is seen in three cases (20%). Nivolumab alone was used in three cases (20%). The median time to onset is eight weeks (SD 17,4), with a quite large variable between only two weeks for the shortest and 17 months for the longest one (11–13). Six patients (40%) suffered from other irAEs, including two with diffuse interstitial pulmonary involvement, including case 1 (2, 3, 5, 13). The main clinical presentation is a distal and painful necrosis mostly on the hands (12/15 cases, 80%) with bilateral involvement (11/15, 73%). Toes were affected only in three cases (20%) (5, 12, 13). The ears and nose can also be affected (five). Interestingly, for six patients (40%), the first clinical phase is described as a Raynaud’s-like syndrome, including case 1 (2, 4, 9, 13). All cases have severe features with grade CTCAE (v5.0) ≥ 3. In each case, a large work-up was realized, as recommended to identified etiologies, with large blood samples and at least an arterial Doppler ultrasound test, sometimes completed with a CT chest or arterial angiography (4). Macrovascular disease is sometimes detected but is not sufficient to explain all the clinical signs, like in case 2. (five) (12). When a nail fold capillaroscopy was performed, it did not reveal any pathological findings, like in case 1 and 3 (4, 5, 8, 9). On immunological tests, ANAs were found in six patients (40%), with a median titer of 1:400 (min-max 80-5200), without any speckled pattern in most of them (3–5, 7, 9). Sometimes specific antibodies were found, like anti-RNA pol-III in one patient, anti-SSa with type III cryoglobulin I in another one, and anti-Jo1 in our case 3 (3, 9). None of the patients reported a preexisting autoimmune disease prior to initiating immunotherapy treatment. Biopsy was rarely performed, probably due to risk of worsening lesions and delaying healing; biopsy results are available for only for three (20%) patients (7, 8, 13). Histopathology revealed both thrombosis and perivascular inflammatory infiltrates including lymphocytes, plasma cells, and neutrophils, suggestive of vasculitis (3, 8, 13).

Most patients were treated with steroids, namely 12/15 (80%), sometimes with high doses of IV pulses (5/15) (33%). Only three patients (20%) received one immunosuppressive or immunomodulating agent (IS/IM): mycophenolate mofetil (MMF), rituximab, and tocilizumab, respectively, for each patient (6,7%) (2, 10). Aspirin and anticoagulation were initiated in five (33%) and three (20%) cases, respectively. Seven patients (47%) received at least one calcium blocker. Iloprost was tried in seven cases (47%), prostacyclin analogs in four (27%), and sildenafil in three (20%). Surgical amputation was necessary for four patients (27%) (2, 4, 8, 12). In case 1, surgical amputation was performed late only after stabilization of the digital necrosis. In all cases, ICI was stopped and no rechallenge was attempted. In most cases, CS are not enough to stabilize the ischemia, even with high doses. In the three patients (20%) treated with IS/IM, two had a complete response: one with MMF and our case 1. after tocilizumab perfusion (10). Rituximab did not prevent the third patient from worsening (2). Most patients with a partial or complete response were treated by iloprost or prostacyclin analogs perfusions (3, 7, 10, 11). Calcium blockers are often associated with variable efficiency. Finally, one patient (6,7%) underwent sympathectomy without any improvement of acral necrosis (4). Oncology assessment shows cancer progression in 5/9 (56%) patients. Two patients experienced a complete response and two achieved stable disease (11%).

The pathophysiology of acral vasculitis could be based on the alteration of the immunological homeostasis with activation of the T-cell population or antibodies forming against self-antigens, possibly against endothelial cells (4). Some authors found that blockage of the PD-1/PD-L1 signal initiates T-cell infiltration of the vascular endothelium and can lead to medium/large vessels; blockage of other signals can lead to PD-1 receptor impairment and induce auto-antibodies against shared antigens between tumor and normal tissue in mice models (4, 14). This hypothesis seems to be supported by the positivity of ANAs in a large portion of patients in our cohort and encourage rapid initiation of high-dose CS (9). Similarly, in the case of steroid-refractory vasculitis, we believe that rapid IS/IM agents must be introduced (3–5, 7, 9). Even Franco et al. described a complete response with MMF; we think drugs with a very short onset of action, like tocilizumab used in case 1, are to be preferred., Plasma exchange or anti-JAK (Janus Kinase) therapy could also be viable options, although these were not tried in our cohort (10). Another mechanism to explain acral vasculitis could be a proinflammatory effect caused by ICIs with endothelial injury that could induce either atherosclerosis lesions or a procoagulable state (4). In the same way, several authors discussed a paraneoplastic (PNS) origin as observed on paraneoplastic acral vascular syndrome (PAVS), mostly associated with lung adenocarcinomas and stomach and breast cancers (4, 7–9, 15). PAVS seems to be due to several mechanisms such as hyperviscosity, hypercoagulability, generalized vasospasm, and spontaneous platelet aggregation (8, 15). Le Besnerais et al, found that, of 100 patients with digital ischemia, there was a significantly higher rate of thrombocytosis on cancers patients, demonstrating an indirect measure of hypercoagulability (15). Interestingly, the clinical presentation of patients in our cohort shows some similarities to features found in PAVS, especially a Raynaud’s phenomenon that preceded digital ischemia and a bilateral and mostly hands involvement (9, 15). ICIs could be triggered by both underlying or a new PAVS. We believe this finding must lead us to try strong vasodilators such as iloprost and stop ICI perfusions earlier. Aspirin may also be useful in reducing the risk of thrombosis (5). However, given the paucity of cases and retrospective data, our conclusions are limited. The absence of uniform diagnostic criteria and histological data in most cases is a damaging point, even if often justified by authors. Similarly, the comprehensive work-up is not homogeneous and not always exhaustive enough to rule out a differential diagnosis to this immuno-related vasculitis. Vascular risk factors like diabetes and smoking have been also reported in some patients who developed digital ischemia (4). This information in clinical cases is not always known and can constitute a bias in the interpretation of our results. Moreover, there is no clear treatment response criteria and a lack of long-term follow up to define the optimal treatment in this situation. Finally, data about rechallenge after vascular irAEs are also missing and contraindication after severe digital ischemia must remain the rule.

## Conclusion

ICI-induced vasculitis can concern small vessels and lead to severe digital ischemia, often in males and preceded by a Raynaud’s like syndrome with mostly bilateral and hands involvement. Clinicians should be careful about acrosyndrome occurring during treatment and closely monitor for the development of digital necrosis in these patients. We believe that quick high-dose corticosteroids associated with vasodilator agents can lead to real clinical improvement. Immunosuppressive agents are sometimes necessary. If acral vasculitis can be considered as a paraneoplastic syndrome, ICIs must be quickly stopped, although data about rechallenge is currently too limited to try this out. Data are still missing and more studies are necessary to optimize management and specify when rechallenge can be discussed. A better understanding of vasculitis physiopathology would also be important in the choice of second-line immunosuppressive treatments.

## Data Availability

The original contributions presented in the study are included in the article/supplementary material. Further inquiries can be directed to the corresponding author.
